# Accumulation of an Endogenous Tryptophan-Derived Metabolite in Colorectal and Breast Cancers

**DOI:** 10.1371/journal.pone.0122046

**Published:** 2015-04-16

**Authors:** Paolo Puccetti, Francesca Fallarino, Antoine Italiano, Isabelle Soubeyran, Gaetan MacGrogan, Marc Debled, Valerie Velasco, Dominique Bodet, Sandrine Eimer, Marc Veldhoen, Georges C. Prendergast, Michael Platten, Alban Bessede, Gilles J. Guillemin

**Affiliations:** 1 Department of Experimental Medicine, University of Perugia, Perugia, Italy; 2 Department of Medical Oncology, Institut Bergonié, Bordeaux, France; 3 ImmuSmol, Pessac, France; 4 Histology and Molecular Pathology of Tumors Laboratory EA 2406, University Bordeaux Segalen, Bordeaux, France; 5 Laboratory for Lymphocyte Signalling and Development, The Babraham Institute, Cambridge, United Kingdom; 6 Lankenau Institute for Medical Research, Wynnewood, Pennsylvania, United states of America; 7 CCU Neuroimmunology and Brain Tumor Immunology, German Cancer Research Center, Heidelberg, Germany; 8 Department of Neurooncology, University Hospital, Heidelberg, Germany; 9 Macquarie University, Faculty of Medicine, Neuroinflammation group, Sydney, New South Wales, Australia; 10 Applied Neurosciences Program, Peter Duncan Neurosciences Research Unit, St Vincent’s Centre for Applied Medical Research, Darlinghurst, New South Wales, Australia; Imperial College London, UNITED KINGDOM

## Abstract

Tumor immune escape mechanisms are being regarded as suitable targets for tumor therapy. Among these, tryptophan catabolism plays a central role in creating an immunosuppressive environment, leading to tolerance to potentially immunogenic tumor antigens. Tryptophan catabolism is initiated by either indoleamine 2,3-dioxygenase (IDO-1/-2) or tryptophan 2,3-dioxygenase 2 (TDO2), resulting in biostatic tryptophan starvation and l-kynurenine production, which participates in shaping the dynamic relationship of the host’s immune system with tumor cells. Current immunotherapy strategies include blockade of IDO-1/-2 or TDO2, to restore efficient antitumor responses. Patients who might benefit from this approach are currently identified based on expression analyses of IDO-1/-2 or TDO2 in tumor tissue and/or enzymatic activity assessed by kynurenine/tryptophan ratios in the serum. We developed a monoclonal antibody targeting l-kynurenine as an *in situ* biomarker of IDO-1/-2 or TDO2 activity. Using Tissue Micro Array technology and immunostaining, colorectal and breast cancer patients were phenotyped based on l-kynurenine production. In colorectal cancer l-kynurenine was not unequivocally associated with IDO-1 expression, suggesting that the mere expression of tryptophan catabolic enzymes is not sufficiently informative for optimal immunotherapy.

## INTRODUCTION

For their own persistence, malignant cells must defy the host’s immune system, a mechanism known as tumor evasion. There is a resurgence of interest in the mechanisms of immune escape by tumors, owing to a growing understanding of the molecular biology of malignant cells, the recognition of the role of the tumor microenvironment, the identification of new therapeutic targets, and the design of several novel immunotherapeutic strategies, among which is targeting immunological synapses to enhance host's immune reactivity [1]. Tumors, in fact, evade otherwise effective T-cell responses—either spontaneously elicited or fostered by therapeutic maneuvers—by exploiting potent endogenous immunosuppressive mechanisms within their local environment, often subjugating and diverting immune tolerance pathways that normally protect healthy tissues from autoimmune damage.

**Fig 1:**
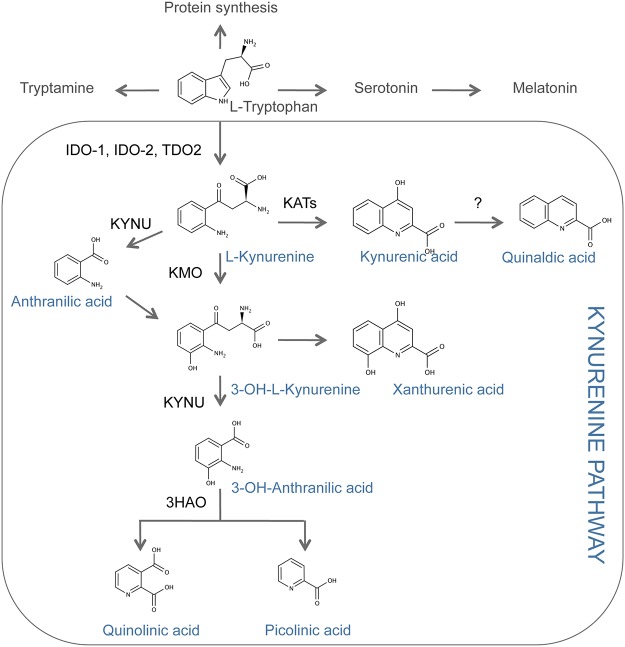
Overview of the tryptophan metabolism and the kynurenine pathway. IDO-1: Indoleamine 2,3-dioxygenase 1. IDO-2: Indoleamine 2,3-dioxygnease 2. TDO2: Tryptophan 2,3-dioxygenase. KATs: Kynurenine amino transferases. KMO: Kynurenine-3-monooxygenase. KYNU: Kynureninase. 3HAO: 3-hydroxyanthranilate oxygenase

In mammals, tryptophan catabolism is a physiological means of preserving immune homeostasis and tolerance—including maternofetal tolerance—and avoiding acute and chronic hyper-inflammatory reactions and autoimmunity [2]. Tryptophan degradation is initiated by three different enzymes, namely, indoleamine 2,3-dioxygenase 1 (IDO-1), its paralogue IDO-2, and tryptophan 2,3-dioxygenase 2 (TDO2; mostly expressed in the liver) (Fig 1). All three enzymes induce biostatic tryptophan starvation that limits lymphocyte expansion, and produce several catabolites, collectively known as kynurenines [3]. l-kynurenine, an amino acid itself, is the first, stable tryptophan catabolite in this pathway. l-kynurenine induces T helper type-1 cell apoptosis [4], and can also act as an endogenous activator of the ligand-operated transcription factor aryl hydrocarbon receptor (AhR), thus altering immune responses [5, 6].

Increased expression of indoleamine 2,3-dioxygenases has been observed in several types of human solid tumors, including colorectal, breast, ovarian, lung cancers and melanoma [7–11], and in hematological malignancies as well, such as acute myeloid leukemia [12] and lymphoma [13]. In colorectal cancer (CRC), IDO-1 overexpression correlates with reduced tumor infiltration by lymphocytes, increased rates of hepatic metastases, and a poor clinical outcome [14]. In skin lymph nodes from melanoma patients, IDO-1 expression is associated with lower survival rates. Recently, TDO2 was shown to be overexpressed in a large panel of tumors [15], with a specific, and crucial role in glioma progression [16]. TDO2 overexpression in high-grade gliomas correlated, with a poor prognosis, implying tryptophan-derived l-kynurenine as an tumor-derived metabolite promoting AhR-driven immune suppression [16].

Experimentally, when expressed by dendritic or cancer cells, both indoleamine 2,3-dioxygenases can suppress tumor-specific immune reactivity [7]. Thus a novel therapeutic approach has been developed to inhibit those enzymes, through the use of 1-methyl-tryptophan (1-MT). The levo-isoform (L-1-MT) blocks IDO-1, whereas dextro-1-MT (D-1-MT), which has been used in clinical trials, preferentially inhibits IDO-2 [17]. Although blockade of tryptophan catabolism using specific enzyme inhibitors may represent a new effective strategy in cancer, the main cellular protagonists—whether host’s or tumor’s—responsible for the enzyme activity and the exact mode of action of 1-MT remains unclear. Perplexities have arisen, in general, as to therapeutic efficacy of IDO-1/-2 inhibitors. Furthermore, measurements of the systemic kynurenine-to-tryptophan ratio is unlikely to be predictive of increased tryptophan degradation at the tumor site [18]. This would require a direct assessment of kynurenine production at the tumor site.

We have developed a new and highly specific monoclonal antibody to detect l-kynurenine within tissue specimens. The antibody was used in semi-quantitative immunohistochemistry (IHC) studies aiming to investigate l-kynurenine production by colorectal and breast cancer specimens directly. Antibody-based detection of kynurenine within tumor specimens might represent a novel strategy to identify tryptophan-degrading tumors and patients most likely to benefit from blockade of immune suppressive tryptophan catabolism.

## MATERIAL AND METHODS

### Antibody development

The experimental protocol for mice immunization was approved by the Animal Care and Use Committee of the University of Bordeaux (Comité d'éthique pour l'Expérimentation Animale, Université de Bordeaux) on December 13th, 2012, under number 50120171-A. For immunization, l-kynurenine was conjugated to bovine serum albumin (BSA) and BALB/c mice (Charles River, Larbresle, France) were immunized three times over a 2-month period. Serum samples were collected 10 days after the last immunization and were assayed by mean of ELISA for the presence and characteristics of anti- l-kynurenine antibodies. After cervical dislocation, spleens from animals with high affinity and specificity antibodies were retrieved and splenocytes were used to generate hybridomas per standard fusion procedures. The resulting hybridomas were grown to confluence, after which cell supernatants were screened by ELISA to identify positive hybridoma clones. Three positive hybridomas were then subjected to limiting dilution. Monoclonal antibodies were obtained from cell culture supernatants and were then purified.

### Enzyme Linked Immuno Sorbent Assay (ELISA)

Maxisorp 96-well plates (Nunc) were coated overnight with conjugated l-kynurenine. Plates were rinsed and blocked with BSA 2,5g/L diluted in PBST for 1 hr at 37°C. For the primary incubation, mice sera, hybridoma supernatants or purified IgG were incubated with increasing concentrations of l-kynurenine or kynurenine analogs conjugated to the same protein carrier (PC). Plates were washed and incubated with HRP-conjugated goat anti-mouse IgG secondary for 1 hr at 37°C. Plates were then exposed to tetramethylbenzidine for 10 min. The detection reaction was stopped by the addition of 2N HCl and optical density was determined at 450 nm.

### Tissue samples

All tissues used in this study were purchased at US Biomax Inc. Tissues were collected under the highest ethical standards with the donor being informed completely and with their consent. All human tissues are collected under HIPPA approved protocols. All samples have been tested negative for HIV and Hepatitis B or their counterparts in animals, and approved for commercial product development. Formalin-fixed and paraffin-embedded colorectal and breast cancer samples were studied as Tissue Micro Array. CO1503 samples were used for colorectal cancer while BR1503 sample was used for breast cancer. Clinical and histopathological data are listed in the S1 and S2 Tables for CRC and Breast Cancer respectively.

### Immunohistochemistry

Immunostaining was done on paraffin- embedded Tissue Micro Array sections (5 μm). After deparaffinization and rehydration, the sections were subjected to microwave antigen-retrieval with pH = 6/9 citrate buffer (Dako, Copenhagen, Denmark). After washes, endogenous peroxidase was blocked using hydrogen peroxide prepared in methanol. Non-specific binding was blocked for 30 minutes with antibody diluent (Dako) supplemented with 5% BSA (ID Bio, France) at room temperature. Sections were then exposed over-night at 4°C with mouse anti- l-kynurenine (clone 3D4-F2–1/1000 dilution) or mouse anti-IDO-1 (Origene Technologies) antibodies in diluent supplemented with 2% Normal Goat Serum (Dako). After washing, envision system (HRP-conjugated polymer backbone linked to secondary antibodies, Dako) and DAB-chromogen were applied (Dako). Tissue sections were counterstained with hematoxylin. Stainings were captured using Tissue Gnostic scanning microscopy. Immunoreactivity was semi-quantitatively estimated and two cores per case were graded as 0 (no staining), 1 (weak staining), 2 (moderate staining) and 3 (strong staining). For both l-kynurenine and IDO-1, an average value for two cores was calculated to define immunoscore.

## RESULTS AND DISCUSSION

**Fig 2:**
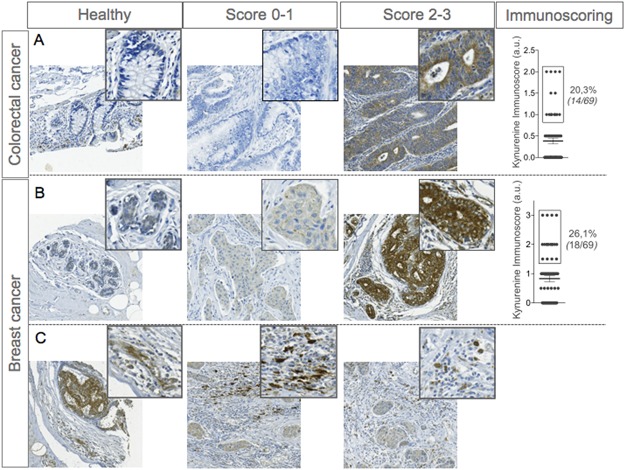
Immunodetection of l-kynurenine in colorectal and breast tumour specimens. **A** and **B**, Representative micrographs of immunohistochemical stainings of paraffin-embedded healthy epithelia and colorectal or breast cancer samples using specific antibodies targeting kynurenine (clone 3D4-F2). On the right panel, graph represents kynurenine immunoscore (obtained from 2 independent TMA cores) with % of Kynurenine positive patients. (**C**) Representative micrographs of kynurenine immunostainings of paraffin-embedded breast cancer microenvironment.

Tryptophan catabolism is known to be involved in tumor progression by favoring immune escape and tumor-induced immune suppression [7, 15, 16]. Inhibition of tryptophan catabolic enzymes represents an attractive therapeutic strategy, aiming at reinstalling an effective tumor-specific protective immunity [19–21]. We investigated the local production of l-kynurenine, the first metabolite produced through the kynurenine pathway (Fig 1), in two different types of human tumors, namely, colorectal and breast cancers (See patients characteristics in S1 and S2 Tables). In attempt to detect l-kynurenine in situ we developed a monoclonal antibody. The antibody was demonstrated to be highly affine and specific for its target (S1 Fig). Colorectal (CRC) and breast cancer specimens were then assayed by IHC on tissue microarrays (TMA). As a control, normal tissue was included for each histotype. Because there occur caveats on removing tissue cores from a whole tumor section—particularly when dealing with non-homogenous staining profiles (as it is the case for kynurenine; S2 Fig)—we evaluated profiles on two independent cores from each tumor specimen. Of the 69 CRC samples tested, 14 (20.3%) stained positive for l-kynurenine (Fig 2A). Because healthy colon epithelial cells were negative, and weak positivity was observed in gut-infiltrating immunocytes, tumor samples were considered competent for kynurenine production when the IHC score was ≥ 1. To confirm the specificity of 3D4-F2 staining, the antibody was incubated with the kynurenine conjugate before staining a colorectal sample found to be positive for l-kynurenine. Pre-exposure of the antibody to the conjugate completely abolished its ability to stain the otherwise positive tumor sample (S3 Fig), demonstrating the specificity of the staining.

l-kynurenine was detected in 18 (26.1%) out of 69 breast cancer specimens (Fig 2B), and because section from healthy epithelial cells showed limited amounts of l-kynurenine; positivity was thus defined as an IHC score ≥ 1.5. In all positive samples, l-kynurenine accumulated in the cytoplasm of tumor cells, in accordance with previous findings that tryptophan catabolic enzymes—IDO-1, IDO-2 and TDO2—are also present in the cytoplasm [16, 17]. Occasionally, l-kynurenine also occurred in stromal cells of the tumoral microenvironment (Fig 2C), which is consistent with the expression of IDO-1/2 by immunocytes and endothelial cells [22, 23]. Perhaps owing to an insufficient number of l-kynurenine-positive samples, no statistically significant association between l-kynurenine immunoscore and clinical data—i.e., tumor grade and size, lymph node invasion and metastases—could be established for either tumor type (Data not shown).

**Fig 3:**
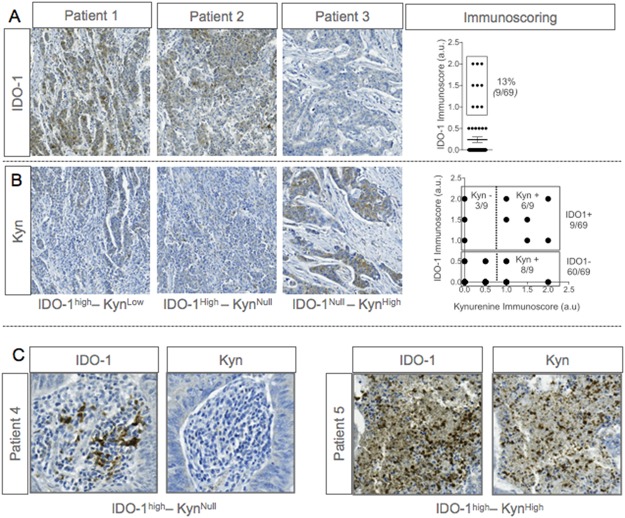
Immunodetection of IDO-1 and l-Kynurenine in colorectal cancer samples. **A** and **B**, Representative micrographs of immunohistochemical stainings of paraffin-embedded colorectal cancer samples using specific antibodies targeting IDO-1 or l-kynurenine. On the up-right panel, graph represents IDO-1 immunoscore (obtained from 2 independent TMA cores) with % of IDO-1 positive patients. On the downright panel, scatter plot represents IDO-1 immunoscore over kynurenine immunoscore (**C**) Representative micrographs of IDO-1 and kynurenine immunostainings of immune cells from paraffin-embedded colorectal cancer samples.

As l-kynurenine production is mostly dependent on IDO-1 activity, we investigated IDO-1 expression in the same cohort of CRC patients using IHC. IDO-1 was up regulated in 9 (13%) out of 69 samples (Fig 3A), a percentage similar to that reported by Gao et *al* [24], but lower than the 39% value found by an earlier study [14], which suggests significant variability among tumor specimens even of the same histotype. Similarly with l-kynurenine detection, no positive correlation could be established between IDO-1 expression and clinical data (grade and size of the tumor, lymph node invasion and metastases; data not shown). When present, IDO-1 was invariably expressed in the cytoplasm of tumor cells, but there were also instances of noticeable expression by cells from the microenvironment [24].

A correlation study of l-kynurenine accumulation and IDO-1 expression revealed that a few patients (3/69) expressed IDO-1 in the absence of l-kynurenine production, (Fig 3B), indicating that even when tumors are IDO-1-competent, they do not necessarily produce l-kynurenine. The same pattern was occasionally observed in infiltrating immune cells, where IDO-1 was expressed and l-kynurenine was undetectable (Fig 3C). In several necrotic areas, IDO-1 and l-kynurenine were highly co-expressed. Conversely, tumor samples with an IDO-1-negative but kynurenine-positive phenotype were also found on TMA analysis. Because IDO-2 or TDO2 are also able to synthetize l-kynurenine, it is possible these enzymes, either alone or in combination, were a major source of the metabolite in those instances [16, 17]. Altogether, these data indicate that IDO-1 expression is not strictly associated with l-kynurenine production in cancers. Further investigation should assess IDO-2 and TDO2 expression in these tumors.

Most observations supporting a putative role for tryptophan catabolism in tumor progression came from studies of IHC-based detection of IDO-1, IDO-2 or TDO2 in tumor specimens [25], on the assumption that any resulting kynurenine production would favor tumor escape mechanisms. Alternatively, measurements of systemic kynurenine-to-tryptophan ratio [26–34] were taken as a predictor of increased tryptophan catabolism activity. Yet, no studies have directly addressed the issue of the source and extent of kynurenine production at the tumor site.

Accumulation of l-kynurenine was documented in this study in several instances, to variable extents. Notably, in CRC, l-kynurenine detection was not strictly associated with IDO-1 expression, and there occurred instances where l-kynurenine was undetectable in the face of IDO-1 expression, or conversely, kynurenine was present in the absence of tumoral IDO-1 competence. These result suggest that, at least in solid tumors, neither measurement of systemic kynurenine-to-tryptophan ratios nor assessment of local IDO-1 competence in the tumor or its environment may be indicative, *per se*, of the occurrence of l-kynurenine as a suppressive oncometabolite. Other kynurenine pathway metabolites such as 3-hydroxyanthranilic acid [35, 36] or cinnabarinic acid [37] may be considered in further studies.

*In situ* detection of Kynurenine in cancer samples offers the advantages to fits perfectly with routine procedures in immunohistochemistry and can be easily added to others markers. Combining this antibody with automat systems could offer the possibility to analyze large cohort of samples and address correlation between *in situ* Kynurenine and tryptophan degradating enzymes (IDO1, IDO2, TDO2) but also clinical data (survival, molecular status, grade, lymph nodes invasion, metastasis, etc.).

Overall, when contextualized to the implementation of oncotherapeutic strategies where the use of biomarkers can help matching patients to an optimal treatment to improve patient outcomes, our study calls attention to the possibility of *in situ* detection of l-kynurenine as a potential biomarker to select patients that are most likely to benefit from blockade of tryptophan catabolic enzymes.

## Supporting Information

S1 Fig L-kynurenine-specific monoclonal antibodies. We developed L-kynurenine-specific monoclonal antibodies to detect the *in situ* production of tryptophan catabolites. Competitive ELISA was used to determine the affinity of three different monoclonal antibodies (mAb)– 5C1-G5, 2E6-F7, and 3D4-F2—for bovine serum albumin (BSA)-conjugated l-kynurenine. Of the three antibodies, 3D4-F2 demonstrated the highest affinity for the conjugate (5 × 10^-10^ M; **A**). The 3D4-F2 antibody did not react with other conjugated kynurenine derivatives, including 3-hydroxykynurenine, anthranilic, kynurenic, quinaldic, xanthurenic, 3-hydroxyanthranilic, or quinolinic acids (**B** and data not shown). Interestingly, limited yet detectable binding of the antibody also occurred with free l-kynurenine (data not shown), indicating immunodominance of l-kynurenine epitopes in the conjugate.
(TIF)

S2 Fig l-kynurenine immunostaining is heterogeneous over a tumour section. Representative micrographs of immunohistochemical stainings of paraffin-embedded colorectal cancer sample using specific antibodies targeting l-kynurenine in two cores regions.
(TIF)

S3 Fig L-kynurenine staining is specific. Representative micrographs of immunohistochemical staining of paraffin-embedded colorectal cancer sample using specific antibodies targeting L-kynurenine previously incubated or not with the antigen—L-kynurenine conjugate.
(TIF)

S1 Table Clinical and histopathological CRC patient’s data. (DOCX)

S2 Table Clinical and histopathological BC patient’s data. (DOCX)
