# Supplementary figures and images for: Accumulation of an Endogenous Tryptophan-Derived Metabolite in Colorectal and Breast Cancers
Source: PLoS One. 2015 Apr 16;10(4):e0122046. doi: 10.1371/journal.pone.0122046 (PMC4400104; doi:10.1371/journal.pone.0122046)

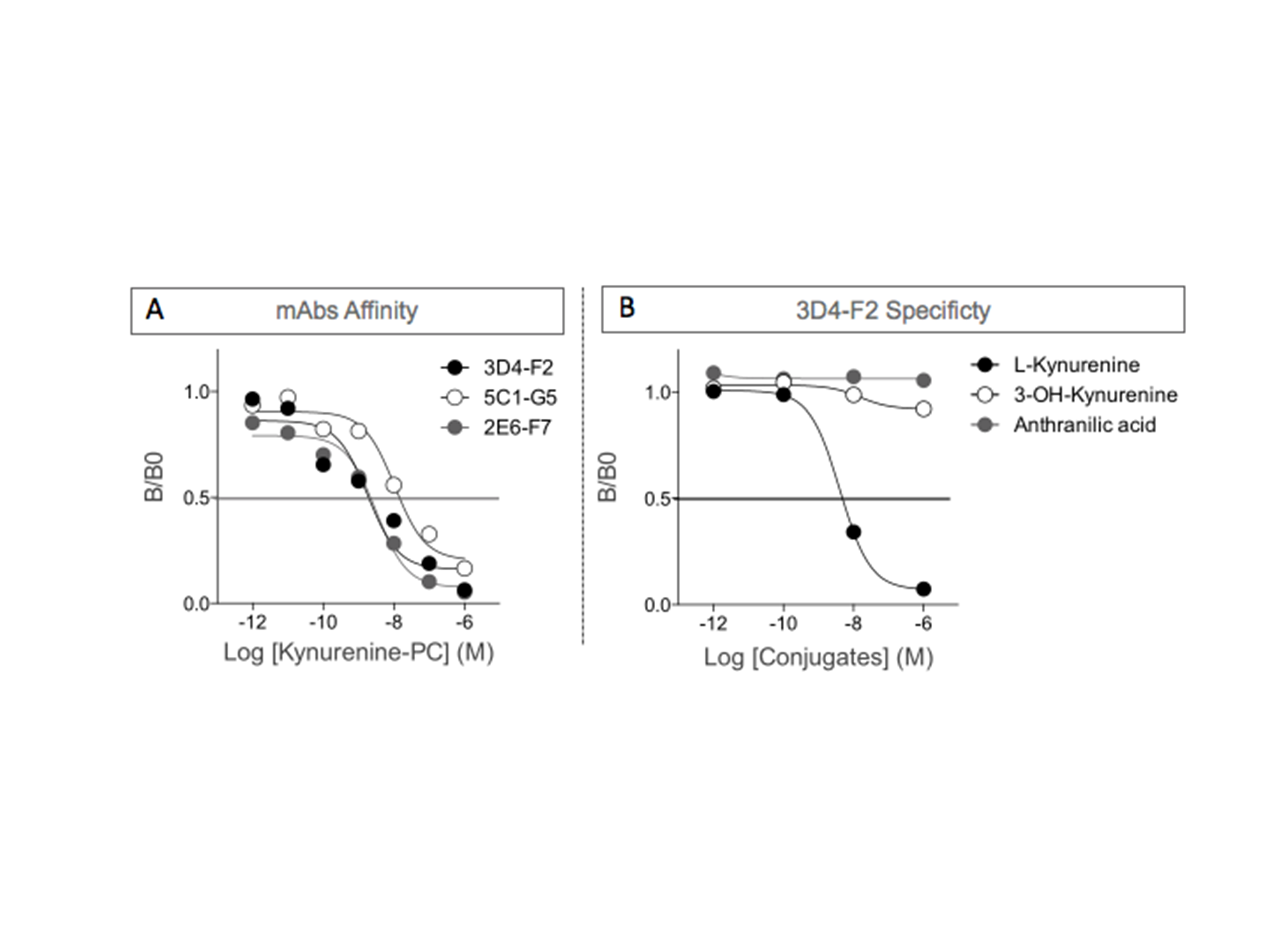

Supplement: S1 Fig — We developed L-kynurenine-specific monoclonal antibodies to detect the in situ production of tryptophan catabolites. Competitive ELISA was used to determine the affinity of three different monoclonal antibodies (mAb)– 5C1-G5, 2E6-F7, and 3D4-F2—for bovine serum albumin (BSA)-conjugated l-kynurenine. Of the three antibodies, 3D4-F2 demonstrated the highest affinity for the conjugate (5 × 10^-10^ M; A). The 3D4-F2 antibody did not react with other conjugated kynurenine derivatives, including 3-hydroxykynurenine, anthranilic, kynurenic, quinaldic, xanthurenic, 3-hydroxyanthranilic, or quinolinic acids (B and data not shown). Interestingly, limited yet detectable binding of the antibody also occurred with free l-kynurenine (data not shown), indicating immunodominance of l-kynurenine epitopes in the conjugate. (TIF) [file pone.0122046.s001.tif]

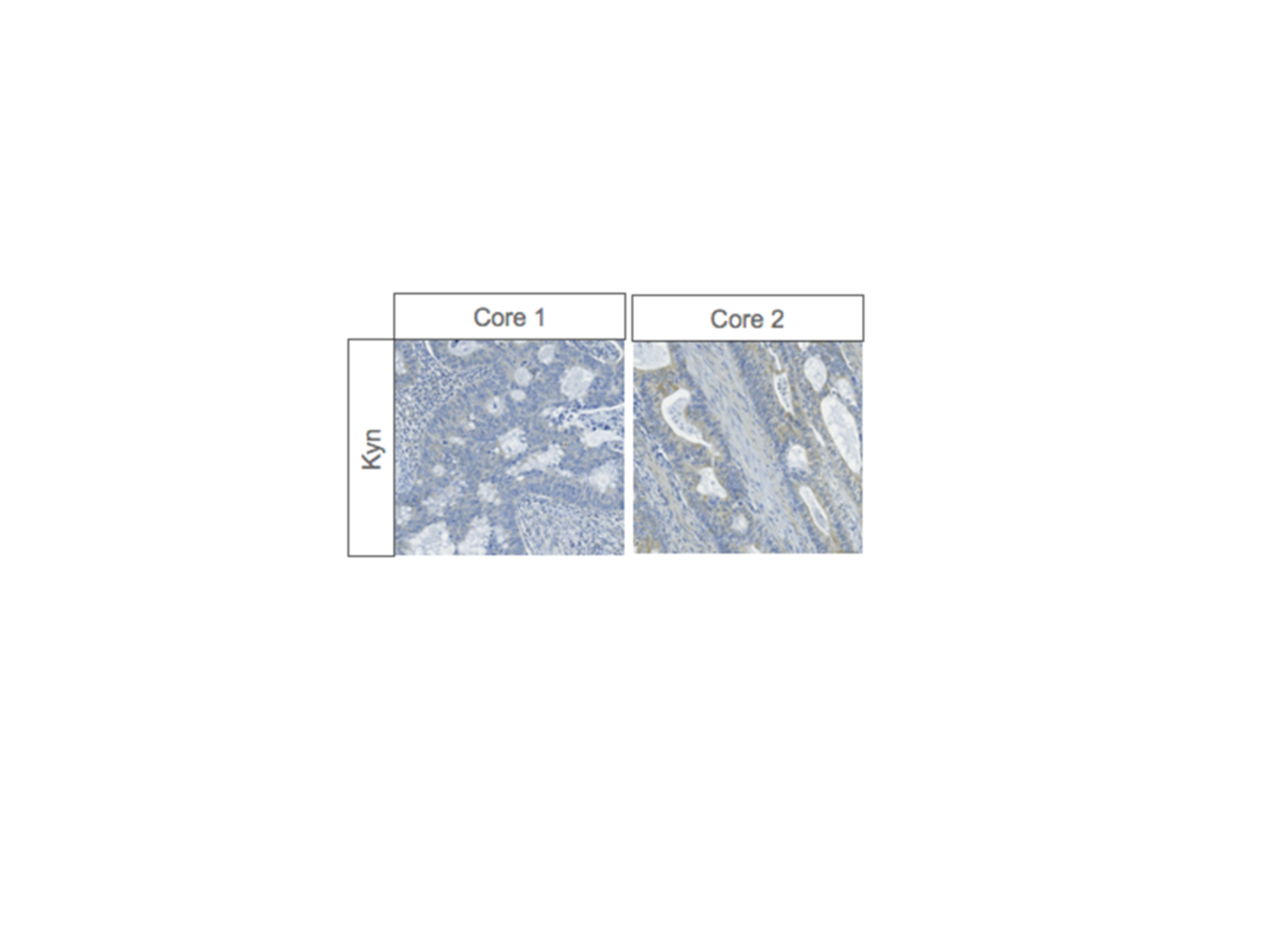

Supplement: S2 Fig — Representative micrographs of immunohistochemical stainings of paraffin-embedded colorectal cancer sample using specific antibodies targeting l-kynurenine in two cores regions. (TIF) [file pone.0122046.s002.tif]

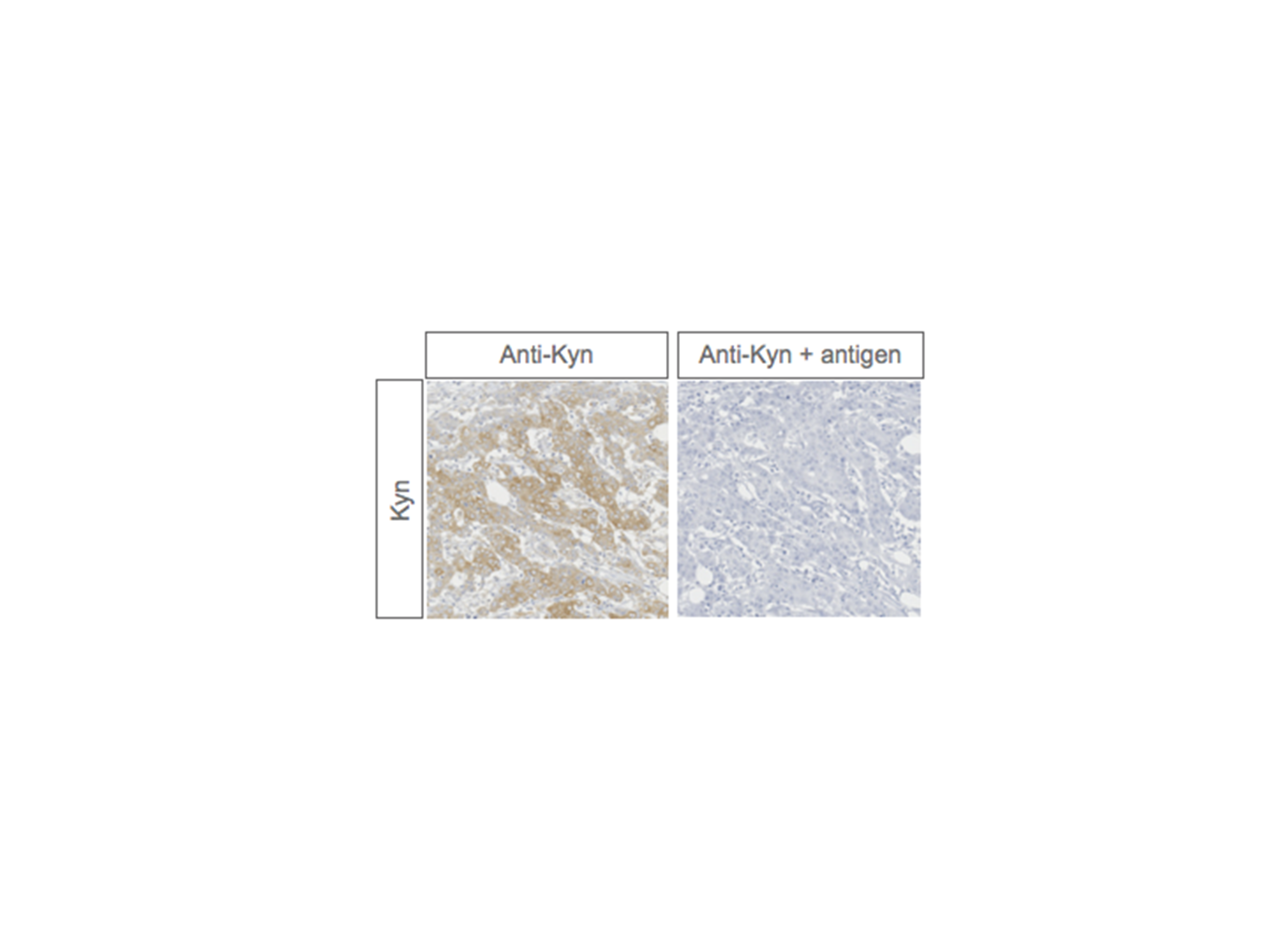

Supplement: S3 Fig — Representative micrographs of immunohistochemical staining of paraffin-embedded colorectal cancer sample using specific antibodies targeting L-kynurenine previously incubated or not with the antigen—L-kynurenine conjugate. (TIF) [file pone.0122046.s003.tif]
