# Supplementary material for: Accumulation of an Endogenous Tryptophan-Derived Metabolite in Colorectal and Breast Cancers
Source: PLoS One. 2015 Apr 16;10(4):e0122046. doi: 10.1371/journal.pone.0122046 (PMC4400104; doi:10.1371/journal.pone.0122046)
Supplement: S1 Table — (DOCX) [file pone.0122046.s004.docx]

**TABLE S1**

| Variable | Number of cases (%) |
| --- | --- |
| Number of patients | *69* |
| Age (y) |  |
| *≤60* | *42 (60,9)* |
| *>60* | *27 (39,1)* |
| Gender |  |
| *Male* | *38 (55,1)* |
| *Female* | *31 (44,9)* |
| Disease stage |  |
| *1-2* | *43 (62,3)* |
| *3* | *23 (33,3)* |
| *4* | *3(4,4)* |
| pT stage |  |
| *1-2* | *3 (4,4)* |
| *3* | *31 (44,9)* |
| *≥4* | *35 (50,7)* |
| Nodal Status |  |
| *N0* | *46 (66,6)* |
| *N1-2* | *23 (33,4)* |
| Histologic grade |  |
| *1* | *20 (28,9)* |
| *2* | *20 (28,9)* |
| *3* | *25 (36,3)* |
| *Missing* | *4 (5,9)* |
| Metastases |  |
| *M0* | *66 (95,6)* |
| *M1* | *3 (4,4)* |
