# Supplementary material for: Accumulation of an Endogenous Tryptophan-Derived Metabolite in Colorectal and Breast Cancers
Source: PLoS One. 2015 Apr 16;10(4):e0122046. doi: 10.1371/journal.pone.0122046 (PMC4400104; doi:10.1371/journal.pone.0122046)
Supplement: S2 Table — (DOCX) [file pone.0122046.s005.docx]

**TABLE S2**

| Variables | Number of cases (%) |
| --- | --- |
| Number of patients | *69* |
| Age (y) |  |
| *≤60* | *62 (89,9)* |
| *>60* | *7 (10,1)* |
| Gender |  |
| *Female* | *69 (100)* |
| *Male* | *0 (0)* |
| Disease stage |  |
| *1* | *6 (8,8)* |
| *2* | *45 (65,2)* |
| *3* | *9(13,0)* |
| *Missing* | *9 (13,0)* |
| pT stage |  |
| *Is* | *7 (10,1)* |
| *1* | *7 (10,1)* |
| *2* | *40 (58)* |
| *≥3* | *13 (18,9)* |
| *Missing* | *2 (2,9)* |
| Nodal Status |  |
| *N0* | *59 (85,5)* |
| *N1-2* | *8 (11,6)* |
| *Missing* | *2 (2,9)* |
| Histologic grade |  |
| *1* | *4 (5,8)* |
| *2* | *49 (71)* |
| *3* | *6 (8,7)* |
| *Missing* | *10 (14,5)* |
| Metastases |  |
| *M0* | *67 (97,1)* |
| *M1* | *0 (0)* |
| *Missing* | *2 (2,9)* |
| Molecular classification |  |
| *Luminal A* | *17 (24,6)* |
| *Luminal B* | *17 (24,6)* |
| *Triple negative* | *16 (23,2)* |
| *Her2-enriched* | *18 (26,1)* |
| *Missing* | *1 (1,5)* |
